# From surviving to thriving: What evidence is needed to move early child-development interventions to scale?

**DOI:** 10.1371/journal.pmed.1002557

**Published:** 2018-04-24

**Authors:** Mark Tomlinson

**Affiliations:** 1 Department of Psychology, Stellenbosch University, Stellenbosch, South Africa; 2 Centre of Excellence in Human Development, University Witwatersrand, Johannesburg, South Africa

## Abstract

In a Perspective, Mark Tomlinson discusses research on early interventions to support child development in developing countries.

Previous research has established that delivering interventions in the first 1,000 days of life improves mother–child attachment [1], contributes to the reduction of health inequities [2], has a significant impact on adult health [3], and is cost-effective [4]. At the global level, the importance of the early years of a child’s life is reflected in high-level World Health Organization (WHO) and United Nations Children's Fund (UNICEF) support [5], the Global Strategy for Women’s, Children’s and Adolescents’ Health (with its focus on not only survival but also children’s ability to thrive and progress successfully into adolescence) [6], and the Nurturing Care Framework to be launched at the WHO World Health Assembly in May 2018. In this week's *PLOS Medicine*, 2 Research Articles present findings from trials conducted in Zambia and Colombia that add to the burgeoning evidence base on the implementation of early interventions designed to improve early child development (ECD) in low- and middle-income countries (LMICs).

## Child development interventions: Mechanism of impact

Peter Rockers and colleagues [7] assessed the impact on ECD of community-based home visiting incorporating health screening and parenting groups. The study was a 2-year follow-up of a cluster-randomised controlled trial conducted in Zambia with the caregivers of children between the ages of 6 and 12 months. The study included fortnightly home visits conducted by child development agents (CDAs) for the first year as well as parenting groups every 2 weeks for 2 years. The intervention significantly reduced stunting (odds ratio [OR] 0.45, 95% CI 0.22–0.92; *p* = 0.028) and was associated with an improvement in child language (β 0.14, 95% CI 0.01–0.27; *p* = 0.039), but there was no impact on other child-development outcomes. While Rockers and colleagues’ study is important and speaks to the difficulties of improving child development in low-resource contexts, one of the key conclusions of the paper is that parenting groups may be a promising avenue for improving physical growth and child development. This of course may be true, but it is more likely that the improvements in stunting were a result of the home visits of the CDAs, and not the parenting groups, given that their visits focused on screening for infections and acute malnutrition and encouraging caregivers to attend routine health services. Without an understanding of the mechanisms involved, drawing conclusions about what component of a complex intervention is the likely agent of change is difficult.

Alison Andrew and colleagues [8] describe the medium-term impacts of an ECD intervention in Colombia. They followed up a cohort that had previously received a psychosocial stimulation intervention integrated into a national cash transfer programme in a cluster-randomised trial that showed benefits for ECD. In the 2-year follow-up study, however, Andrew and colleagues found no impact at age 5 years on any of the outcomes assessed (cognition, language, school readiness, executive function, and behaviour) [8]. The authors hypothesise that one of the reasons for the null finding was that the original effects on child development were too small to be sustained—i.e., that they ‘faded out’.

The concept of fade out is of particular relevance for understanding long-term impacts of early interventions. A recent meta-analysis on childhood interventions showed a steady decline in program effect over time that was observed regardless of the duration of the intervention or when it began [9]. As Andrew and colleagues correctly point out [8] ‘fade out’ also needs to be interpreted in the light of what has come to be known as the ‘resurrection effect’ [9]. That is, early effects that disappear in early or middle childhood (as well as in adolescence) may re-emerge much later in life. However, it may also be the case that early effects may disappear and that sustaining positive findings may be dependent on subsequent quality schooling and life experiences. Given the level of adversity in many LMICs, for early investments to remain productive, families and children will require subsequent access to quality environments such as day care and preschools [9]. In contexts of high risk and adversity, the impact of early interventions may be more durable when they are built upon by interventions during later years [9]. Finally, our understanding of pathways and mechanisms and how the dose, timing, and nature of adversity impact on outcome across the life course is limited [10]. While early intervention is essential and foundational, it is not an inoculation against later developmental disruption. A more sophisticated understanding of mechanism of change coupled with a life-course perspective is key. With a better understanding of mechanism, pathways, and dose, we will be in a better position to determine what kinds of follow-up interventions may be necessary beyond the early years for children with significant levels of cumulative biological or psychosocial risk exposure, in order to maintain and build on early gains [11,12].

## Scaling up early interventions

Despite significant current global health focus on scaling up interventions, knowledge is limited about scaling up programmes in ECD. In the Zambia study, one of the conclusions is that scale-up efforts would likely require a delivery platform integrated into existing structures [7]. Unfortunately, in a low-resource setting such as Zambia, the intervention described by Rockers and colleagues is simply not scalable. The intensive nature of the intervention, including home visits and parenting groups, is beyond the means of all LMICs. The intervention in the Colombia study, on the other hand, was integrated from the start within a national programme [7]. One of the explanations the authors proffer for the null results are concerns with extrapolating findings from efficacy trials (from which much of the current evidence comes) to interventions implemented at scale. This intervention was, however, always an integrated one implemented at scale that had positive outcomes (albeit small), and any compromises were likely there from start. Having said that, the acknowledgment that it may have been worthwhile to hire local supervisors and to increase the frequency of supervision [7] is illustrative of the urgent need for research that attempts to understand what is needed for successful scaling up—above and beyond programme content. When scaling up programmes, the ‘soft’ elements, such as recruitment, training, supervision, and accountability, are often the first to be dropped or reduced in frequency [13].

Looking to the future, the ECD field requires rigorous implementation science research that examines the best models of recruitment, training, and supervision (in addition to programme content) to achieve impact. Finally, the high levels of poverty and developmental risk that persist across the life course in many countries make it imperative that longitudinal cohorts are established in diverse contexts in order to facilitate more informed decisions about the best mix of early and later investments.

## Abbreviations

CDA: child development agent
ECD: early child development
LMIC: low- and middle-income country
OR: odds ratio
UNICEF: United Nations Children's Fund
WHO: World Health Organization
